# Editorial and Miscellaneous

**Published:** 1860

**Authors:** 


Prof. John C. Dalton's late work on Physiology is receiving even more attention from our European medical savans than at home. We hail these indications of the appreciation of our American medical authors, as proof that a better day of international comity among medical men begins to dawn. In the foreign notices of the late books of Professors Dalton, Flint, Gross, Draper, and others, it can no longer be a mooted question over the water, "Who reads an American book?"—*Med. Gazette.*

